# Medical Botany

**Published:** 1828-01-01

**Authors:** 


1. *Medical Botany.* By JOHN STEPHENSON, M.D. and JAMES MORSS CHURCHILL, F.L.S. Nos. IV. V. VI. for April, May, June, 1827.
2. *Flora Medica; containing Botanical Descriptions, Natural History, Chemical Properties and Analysis, Medical Properties and Uses, &c.* Edited by a MEMBER OF THE LONDON COLLEGE OF PHYSICIANS, F.L.S. and assisted by several Members of a Botanical Society. No. I. Nov. 1827. London, Callow and Wilson. Octavo, pp. VIII.-16. Six coloured Plates.

SOME of our readers may recollect that, in a former notice of the first of these publications, we commented, at some length, on the importance of Medical Botany, as an accessory science of medicine, and pointed out some causes for the little attention and cultivation it has received in modern times. We likewise expressed our conviction, that its value was already beginning to be better appreciated; and, as if to realize our anticipations of its speedy revival, another work has since started into existence, the consideration of which we must defer, until we have fulfilled our promise of resuming the notice of the subsequent numbers of Messrs. Stephenson and Churchill's publication.

No. IV.—APRIL, 1827.

PLATE XIII. well represents CONIUM MACULATUM—*Common or Spotted Hemlock*, for which, we believe, other plants, of the same natural family, are often mistaken by ignorant persons; and it is, perhaps, only by such a supposition, that the contradictory statements of authors, respecting its medicinal and deleterious qualities can be reconciled. Some assert that the root is highly deleterious, and others that it is innocuous at all seasons. Störck states that the sliced root yielded a bitter acrid juice, a drop or two of which, applied to his tongue, rendered it painful, rigid, and so much swelled, that he could not speak; whilst our authors, whose experience coincides with the accounts of other experimentalists, inform us that, "having gathered a considerable quantity of the root in March, we ascertained, contrary to our expectation, that its odour was not so strong as that of the few leaves which were springing from it; and after chewing a drachm, we could discover no acrid power, and the taste, instead of being bitter, was sweet, and much resembled the flavour of a raw parsnip." Orfila, in April, gave an ounce and a half of the fresh root to a dog. Forty-eight hours afterwards, he had experienced no ill consequences, and the following day he was only somewhat dejected. Of the beneficial effects of hemlock in cancerous tumours, we once met with a remarkable example in an old woman, who had likewise diseased valves of the heart. The tumour had existed ten or fifteen years, and was of considerable size. It frequently became very painful and irritable, but was always relieved by a strong decoction of the plant, used as a fomentation.

"On painful sores of a scrofulous kind; on ulcers which remain in many irritable constitutions after the use of mercury; on some malignant sores, especially such as are met with on the tongue; on indurations of the breasts, and of the testes, it frequently exerts a most salutary power. It also allays morbid irritability of the system, and is given with marked advantage in pertussis, or hooping-cough, and in those pulmonary diseases which frequently follow inflammation of the thoracic cavity. Chronic rheumatism, also, and anomalous pains of the muscles, are often benefited by its use."

To ensure the due effects of the plant, our authors judiciously recommend the powder of the leaves, gathered in June, just as it commences flowering. The extract, they remark, "can scarcely ever be relied upon, from the carelessness observed in its manufacture." There is, however, another manipulation resorted to by the manufacturers of this and other extracts, which, as it increases the quantity of the product at the expense of its efficacy, cannot be too generally known, nor too widely exposed. The practice we allude to, is that of macerating the bruised plant in water, until the process of fermentation has decomposed the vegetable matter, and the mass is far advanced towards putrescency, after which it is expressed, and evaporated in the usual manner. It is no wonder that extracts thus prepared should prove uncertain and inefficacious; but, were we not certain of the fact, we confess we could scarcely credit, that respectable men should knowingly vend such deteriorated articles, to the great detriment of the lives and health of their fellow creatures, for the consideration of the paltry additional profit they derive from the process.

PLATE XIV. CITRUS AURANTIUM—*The Seville Orange,* furnishing the apothecary its peel, orange flower-water its perfume, and curaçoa its flavour, may be passed without comment, as likewise may—

PLATE XV. OLEA EUROPÆA—*The Olive Tree,* which supplies physic and the table with its oil.

PLATE XVI. ANAGALLIS ARVENSIS—*Scarlet Pimpernel,* furnishes the subject of a beautiful plate; but we scarcely think that a plant, of which two or three drachms of extract were required to destroy the life of a dog, ought to have been introduced into the list of poisons, without sufficient proofs of deleterious effects on the human frame. Abounding, as the work generally does, with quotations from works of general literature, we wonder at the omission of a passage in a satire of the late lamented Premier's, alluding to the property this plant, the "poor man's weather-glass," as it is commonly denominated, possesses, of closing its flowers on the approach of rain.

——————" The Anagallis, prescient flower,
Shuts her soft petals at the approaching shower."—*New Morality.*

As well as the more sentimental expressions of the gentle Hurdis—

————————" With tender sense,
The Pimpernel, which to the humid morn,
Ere yet the shower-shedding cloud appears,
Its bosom closes, and presages wet."

No. V.—MAY, 1827.

PLATE XVII. SOLANUM DULCAMARA—*Woody Nightshade, or Bittersweet,* is correctly represented, but varieties are not uncommon. Several original cases of poisoning by the berries of this shrub, are related in a communication from Mr. Wheeler, of Bayswater. The symptoms which occurred in two children, about an hour after partaking the deleterious repast, were "the most excruciating pains in the whole course of the intestines, attended with great heat in the throat and chest. They could not bear the slightest pressure on the abdomen, and suffered much from nausea, thirst, and prostration of strength." The treatment resorted to was the hot-bath, an emetic, which dislodged the contents of the stomach, leeches to the abdomen, a large dose of calomel, followed by a mixture of castor-oil, manna, and laudanum, "in proper proportions." Injections of beef-broth were also administered. A repetition of the leeches, with mild purgatives and emollient drinks, completed the cure. In a third case, violent vomiting and purging, with contraction of the abdominal muscles, were superadded to the symptoms presented in the former two. Two others proved fatal, but no examination of the bodies was made.

It is remarkable that these berries, so deleterious to the human frame, are eaten with impunity by many animals. Bittersweet is said by various authors to promote all the secretions: some ascribe to it violent cathartic properties, whilst slight narcotic qualities not unfrequently attend its employment. We need scarcely repeat Dr. Bateman's opinion, that "one of the most effectual remedies for lepra, under all its varieties, is the decoction of the leaves and twigs of the Solanum Dulcamara."

Our authors have not noticed the preparation of Solanine from the berries of this species and the S. Nigrum, probably on account of the failure experienced by some who have repeated the process of the discoverer, M. Desfosses. See Majendie's Formulaire.

Plate XVIII. introduces Digitalis Purpurea—*Purple Foxglove*, one of the most useful and generally employed of our indigenous vegetables. In some of the western counties, as well as in Germany, it is called Fingerhut, from which appellation Fuchsius derived the scientific name it bears. Its remarkable power of controlling the pulse, and removing effusions, probably by restoring the balance of the circulation, will doubtless long entitle it to an important rank among vegetable remedies.

Plate XIX. Paris Quadrifolia—*Herb Paris*, a plant of somewhat rare occurrence, is introduced, as we suppose, on account of the emetic qualities of the root, which several reputable authorities satisfactorily attest it to possess, in the dose of one or two scruples, and recommend it as a substitute for ipecacuanha; but its rarity must preclude its extensive employment as a succedaneum; as well as diminish the chance of much danger, resulting from its deleterious qualities. It occurs, however, in many woods in the midland counties.

Plate XX. Tussilago Farfara—*Colt's-foot*, admirably depicted from the pencil and graver of Clarke, has been accounted excellent for coughs and pulmonary complaints from all antiquity. Galen, Pliny, and Dioscorides, recommend the smoke of the dried leaves to be inhaled through a funnel, and it is still in popular use as a substitute for tobacco. Our authors, regardless of these authorities, consider it an unnecessary and useless appendage of the Materia Medica.

No. VI—JUNE, 1827.

Plate XXI. Helleborus Fœtidus—*Fetid Hellebore*, or *Bear's-foot*. The letter-press accompanying this plate, representing a second species of hellebore, presents us with little information that is novel. It was once extensively employed in regular practice for its anthelmintic properties, expelling lumbrici, according to the testimony of respectable authors, with more certainty than almost any other medicine; and it is still commonly used by the vulgar for the same purpose, who give one digit of a leaf to a child a year old. It acts either as an emetic or cathartic, according to the dose administered. The principal value of a knowledge of this plant in the present day, depends upon the deleterious effects its improper exhibition sometimes occasions. The treatment required in such cases must be the same as for H. Niger.

Plate XXII. Arum Maculatum—*Common Arum, Wake Robin*, or *Cuckoo-pint*, furnishes another beautiful subject for pictorial representation. In a recent state, the white fleshy roots of this plant are extremely acrid, occasioning, when tasted, an insupportable sensation of burning, which lasts several hours. From Bulliard's relation of the cases of three children poisoned by eating it, we gather, that it causes inflammation of the whole tract of the digestive tube. Exhibited medicinally, it powerfully stimulates, and promotes all the secretions; to obtain which effects, the root must be used fresh or recently dried, as its acrid properties are soon dissipated by keeping.

Plate XXIII. Asarum Europæum—*Common Asarabacca*, a plant possessing strong emetic and cathartic qualities, was much employed before the introduction of ipecacuanha. Its chief use in the present day is as an errhine, a small pinch of the powdered leaves occasioning an abundant flow of mucus, and sometimes blood, from the nostrils.

Plate XXIV. Rosmarinus Officinalis—*Common Rosemary*, derived from *ros* and *marinus*, instead of *Rosæ Mariæ*, as the author of the *Catholic*

*Friend*, zealous for the honour of the monkish botanists, supposes, may be consigned to poets, lovers, and the grave, without detriment to our readers.

"Rosemary is for remembrance
Between us daie and night;
Wishing that I might alwaies have
You present in my sight."

We can only afford space for a list of the contents of the four following numbers.

No. VII. Rheum Palmatum; Iris Florentina; Tormentilla Erecta; Aconitum Napellus.—No. VIII. Viola Odorata; Cassia Senna; Papaver Rhæas; Acorus Calamus.—No. IX. Gratiola Officinalis; Momordica Elaterium; Œnanthe Crocata; Geum Urbanum.—No. X. Nicotiana Tabacum; Anthemis Nobilis; Morus Nigra; Lavandula Spica.

In closing this analysis, we regret that our duty to the public should make it necessary to remark, that the selection of subjects, and the information adduced in the latter numbers of this splendidly executed work, do not deserve the high commendations we passed upon the first specimens. Too many plants, of little use or doubtful properties, are needlessly introduced, serving to extend the work, and materially increase the expense, without enhancing the value to the purchaser: indeed, we think that several of the most beautiful specimens of the draughtsman's skill might be advantageously substituted for less conspicuous subjects, especially the poisonous fungi, of which none have yet been figured. Should the present system of giving only one or two important plants in each number be persevered in, we see little probability of the work being completed within a reasonable compass or time; but, we trust, the hints we here throw out will not be lost in the proper quarter.

We now turn to the second candidate for public favour, the Flora Medica, edited, as the title assures us, by "A Member of the London College of Physicians, F.L.S. and assisted by several Members of a Botanical Society." This work, published like the former, in monthly numbers, each containing six coloured plates, is pledged to be completed in twenty-eight numbers. Its plan not only embraces the strictly medical department of botany, but likewise professes to give an illustrative explanation of the Linnean system, as well as a list of botanical terms and definitions; and this, too, at half the expense of the other Medical Botany. The plates, though not so minutely accurate, are, for the most part, striking representations of the plants, and are coloured with great care and correctness. The Almond, Mezereon, and Mallow, deserve considerable praise. The letter-press, however, presents many sad specimens of an incompetent hand: such as induce us to pause before we credit its emanating from such learned persons as the title-page asserts. In the introductory pages we find Dioscorides and Galen styled *Roman* botanists: the *botanical* writings of Boerhaave and Bock are ranked among the *most* valuable, whilst those of Ray, Fuchsius, and a host of eminent men, are unnoticed. In the physiology many exploded notions are retained; whilst typographical errors, of a nature that could escape no one acquainted with the subject, disgrace almost every page. Among others, we have marked, p. ii. mar*a*tima; p. iv. li*b*iaceous; p. vi. ba*rr*a, (bacca); p. vii. *Anthemum;* Anethum, we suppose, for as the plant is pentandrous, it cannot be Anthemis. P. viii. the Genus Ros*æ*, &c. &c. We likewise observe that the Almond is stated to flower "early in February," and the Mezereon "early in January or February;" circumstances that indicate a want of attention in the writer, when compiling from the productions of a more southern climate. If the publishers persevere in their undertaking, we would urge them to cancel the introductory pages altogether. A set of good plates, at the price of this work, is much wanted, and it will not be difficult to find competent persons to conduct the letter-press department.

